# Influenza immunization among Chinese seniors: Urgent calling for improving vaccination coverage, education, and research

**DOI:** 10.1002/agm2.12103

**Published:** 2020-03-27

**Authors:** Xin Li, Sean X. Leng

**Affiliations:** ^1^ Department of Geriatrics The Second Hospital of Tianjin Medical University Tianjin China; ^2^ Division of Geriatric Medicine and Gerontology Johns Hopkins University School of Medicine Baltimore Maryland USA

It is with great pleasure that we provide this commentary with a focus on influenza vaccination for an expert consensus entitled “Recommendations for influenza and *Streptococcus pneumoniae* vaccination in elderly people in China” to be published in this issue of *Aging Medicine*.^1^ Influenza is a major global public health burden with pandemic threat. Seasonal influenza infection is responsible for 3‐5 million severe illness cases and 290 000‐650 000 respiratory deaths annually worldwide.^2^, ^3^ According to the Centers for Disease Control and Prevention (CDC), influenza affects 5%‐20% of the population each year in the United States.^4^ It is estimated that influenza causes 226 000 excess hospitalizations, 25 000‐69 000 deaths, and US $87 billion excess health‐care cost with over 600 000 life‐years lost annually.^5^, ^6^ Among all infectious diseases, influenza is foremost in its age‐related increase in serious complications, leading to hospitalization, catastrophic disability, and death in older adults.^7^, ^8^ Moreover, influenza frequently causes exacerbation of many chronic conditions that are common in older adults, including cardiovascular diseases,^9^, ^10^ further indirectly impacting senior health and mortality. In fact, over 90% of influenza‐related mortality occurs in persons aged over 65 years.^11^ In the United States, influenza and its secondary pneumonia are the fourth leading cause of death in this population.^12^ Therefore, prevention and treatment of influenza in older adults have become a major public health priority.

Like many preventable infectious diseases, immunization is the main strategy for influenza prevention in addition to the universal contact‐ and airborne‐precaution measures. In the United States, annual influenza vaccination is recommended for all adults aged 50 years and older. The annual vaccination is overall efficacious, with estimated risk reduction of 50%‐70% for influenza infection in young adults and less robust risk reduction in older adults.^13^, ^14^ This reduced efficacy in older adults is thought to be due to immunosenescence and common health conditions, such as frailty.^15^ In recent years, a new generation of influenza vaccines, including high‐dose (HD) and adjuvanted ones, have been approved by the US Food and Drug Administration (FDA) for older adults in the United States and elsewhere, in addition to the standard dose trivalent inactivated influenza vaccine (IIV3).

As Chen et al^1^ point out in the expert consensus paper, influenza vaccination coverage for older adults in China is extremely low. This is in the context of the largest and fastest growing older adult population,^16^ leaving the majority of Chinese seniors unprotected by vaccination against influenza. Historically, China has classified vaccines into two categories. The first‐category vaccines are mostly for childhood immunization, such as measles, mumps, and rubella; diphtheria, tetanus, and pertussis; varicella; and Bacillus Calmette‐Guerin. The Chinese authorities have mandated that these first‐category vaccines be given to every child born in China, free of charge. The second‐category vaccines, influenza vaccine included, are mostly for adult immunization. Immunization with the second‐category vaccines is not mandated and immunization cost is not covered by government‐sponsored health insurance. As such, childhood immunization is nearly universal in China as it should be. For example, two recent studies report Bacillus Calmette‐Guerin vaccination coverage in children in China's Zhejiang Province to be from 88.3% to 98.7%.^17^, ^18^ Influenza vaccination coverage for Chinese seniors, however, is a far cry from optimal and anything but acceptable.

There are a number of other reasons for extremely low influenza immunization coverage for seniors in China, though. In the United States, most older adults recognize the importance of influenza vaccination and proactively get vaccinated each influenza season when the vaccine is available. Their health‐care providers also make the annual influenza immunization as a priority health‐care quality measure of prevention and ensure that their elderly patients are vaccinated in a timely fashion or at the point of care. To make it more convenient, influenza vaccines are available at local pharmacies, community senior centers, churches, and long‐term care facilities where older adults can get vaccinated free of charge and without requiring a prescription. Health‐care providers themselves are required to be immunized at most health‐care facilities each year in order to minimize potential risk of spreading influenza to their elderly patients and enhance herd immunity. Influenza vaccine manufacturers have built infrastructure and mechanisms for vaccine production and delivery to ensure adequate and timely vaccine supplies across the country. In China, however, the public is largely unaware of the significant adverse health impact from influenza, considering it as part of the “winter blue.” Most Chinese seniors do not recognize annual influenza immunization as an important and effective measure for influenza prevention and, therefore, hesitate to get vaccinated. Similarly, many Chinese health‐care providers often equate influenza as the “common cold” and do not acknowledge its disproportionately devastating consequences to older adults. They typically have a busy clinical practice schedule and lack of adequate effort or attention for preventative measures, including influenza immunization. In addition, vaccine administration is not widely available in the community and is only allowed at hospital facilities and CDC local offices. Recent vaccine scandals, such as vaccine delivery after its expiration by some domestic manufacturers, have further eroded public confidence in influenza vaccination in China.

To address these challenges, a roadmap is proposed for working towards universal influenza vaccination coverage for seniors and those who need vaccine protection in China (Figure 1). In this roadmap, four pillars of action at the national and local levels are proposed: (a) policy, (b) education, (c) vaccine supply and quality, and (d) innovation and research. Critically important actions in policy include, but are not limited to, revamping vaccine classification and enacting an influenza vaccination mandate for seniors and those with chronic conditions who are immunocompromised and need vaccine protection as well as providing an insurance payment to cover influenza vaccination cost. It is encouraging to note that the municipal governments of Beijing and Shanghai have recently enacted a policy to provide insurance coverage for influenza vaccination for seniors living in their respective municipal areas. As cited by Chen et al,^1^ vaccination coverage among seniors in Beijing has reached close to 50%.^19^

**Figure 1:**
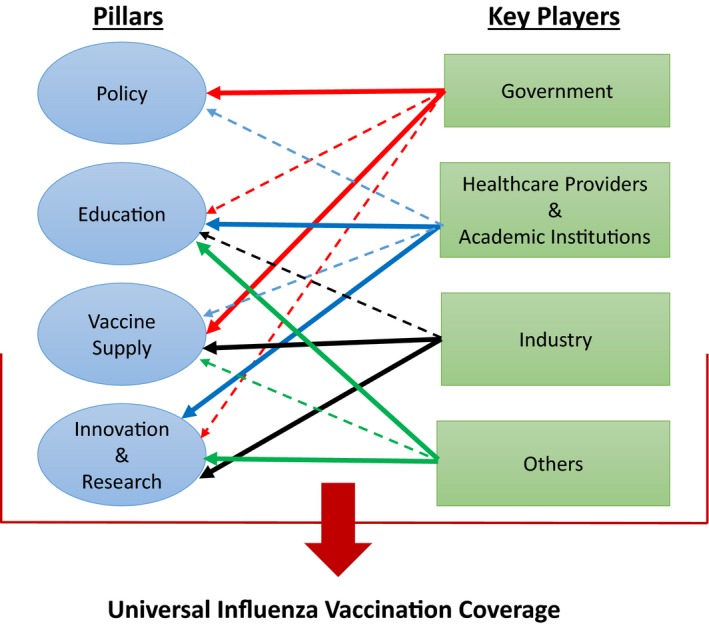
A roadmap towards universal influenza vaccination coverage for seniors and those who need vaccine protection in China.

Education of both health‐care providers as well as the public is also critically important. The expert consensus paper published in this issue of *Aging Medicine* may serve as an initial step towards educating providers in the senior health‐care sector and aging research community alike. However, much of the work remains to be done. For example, most health‐care providers likely recognize the protective effect of influenza immunization against influenza infection and its serious complications and death. However, few are aware of the benefit of influenza vaccination against chronic conditions that are common in older adults. In addition, educating the public is a daunting task and a roadblock that has yet to be tackled.

Needless to say, universal influenza vaccination coverage requires an adequate amount of quality vaccine supply in a timely fashion. Policies and regulations on vaccine manufacture and delivery will need to be established and enforced to ensure vaccine quality and supply. The new generation of influenza vaccines from overseas should also be considered before these vaccines can be produced domestically with quality assurance. This pillar is particularly important to reestablish public confidence since the recent vaccine scandals stated above.

Last but not least is the pillar of innovation and research. After all, it is the effort of innovation and research that has led to the development and FDA‐approved clinical application of the new generation of influenza vaccines described above. In China, the pillar of innovation and research will need to be built from the ground up with special consideration of the following three aspects.

1. There is an urgent need to establish a comprehensive, nationwide influenza surveillance mechanism or network to monitor and report influenza epidemic activity with laboratory diagnosis of influenza infection and confirmation of specific virus strain for each influenza season across China. Such an influenza surveillance mechanism/network will provide important, real‐time information about influenza epidemic activities across different regions for both clinicians as well as researchers and should be instrumental for monitoring other respiratory infections, such as COVID‐19, once it is established. In the United States, while influenza is not listed as a national notifiable condition by the CDC (this list is updated annually, https://wwwn.cdc.gov/nndss/conditions/notifiable/2020/; influenza‐associated pediatric mortality is a reportable condition on the list), the CDC has established a nationwide influenza surveillance network (http://www.cdc.gov/flu). This surveillance network automatically sends out weekly updates about influenza epidemic activities along with a wealth of other helpful information by email to physicians, researchers, and anyone who is interested and has signed up for it. The World Health Organization (WHO) has also established the Global Influenza Surveillance and Response System (GISRS) or FluNet for worldwide influenza surveillance and monitoring (https://www.who.int/influenza/gisrs_laboratory/flunet/en/).
2. For decades, IIV3 had been the only vaccine recommended for older adults in the United States until recently when HD IIV3 and an adjuvanted vaccine were approved by the FDA. As stated above, the current influenza vaccine is only about 50%‐70% efficacious for influenza prevention in young adults and even less effective in older adults. The virus undergoes frequent mutations in its segmented RNA genome that cause “antigenic drift,” requiring annual updates of the vaccine strains as recommended by the WHO. When vaccine strains were not matched to the virulent influenza virus strain in the circulation, the vaccine was even less effective in those “mismatch” seasons. If the virus undergoes major segmental genome reassortments leading to “antigenic shift,” particularly reassortments with strains in its animal reservoir (eg, bird, swine), such a new viral strain evades herd immunity altogether and causes pandemic influenza, as happened in the pandemics of 1918, 1957, 1968, 1977, as well as in the H5N1 (bird flu) outbreak in early 2000s and the swine flu pandemic in 2009. Moreover, the traditional egg‐based vaccine manufacture is labor‐intensive and time‐consuming and could lead to vaccine shortage in some years. There is evidence suggesting that the vaccine strains may undergo antigenic change or modification during the egg‐growth phase, further reducing vaccine efficacy. To search for more efficacious influenza vaccines with less time‐consuming and more reliable manufacturing platforms, influenza vaccine research and innovation have been intensified over the past decade in the United States and elsewhere. In addition to the HD IIV3 and adjuvanted vaccine stated above, there are quadrivalent‐IIV, recombinant‐influenza, live‐attenuated‐influenza, and cell‐based‐influenza vaccines in the development pipeline. In 2017, the National Institute of Allergy and Infectious Diseases (NIAID) of NIH launched the universal influenza vaccine initiative, aiming for an influenza vaccine that conveys over 75% protection against symptomatic disease caused by all influenza A plus/minus influenza B viruses in all populations (including older adults) and that such protection should last longer than 12 months.^20^ In addition to the development of novel and more efficacious influenza vaccines, China can lead the research effort on efficacy assessment of newly developed influenza vaccines. With a large unvaccinated “naive” population, China is in a unique position with the capability of conducting not only double‐blind placebo‐controlled vaccine trials and comparative effectiveness vaccine studies, but also in‐depth studies of vaccine‐induced immunity agaist influenza as well as age‐related immunosenescence.
3. Host factors are the third aspect that is equally critical and deserves more attention. If the host fails to mount an adequate immune response or refuses to get vaccinated, it is irrelevant no matter how good the influenza vaccine is. Immunosenescence that occurs during aging is believed to be a major host factor responsible for the impaired immune protection of influenza vaccination in older adults, further review of which is beyond the scope of this article.^21^, ^22^, ^23^ Age‐related conditions, such as frailty, as well as sex and gender are other important clinical, biological, and social host factors that have significant impact on influenza vaccine immune response and clinical protection.^15^, ^24^, ^25^ Emerging evidence suggests that stem cell therapy may enhance immune response to influenza immunization in aging frailty.^26^, ^27^ Innovation and research will help develop strategies to address these and other host factors. Cutting‐edge geroscience research may pave the way for the development of such interventional strategies.^28^, ^29^

Government agencies, health‐care providers, academic institutions, and the vaccine manufacturing industry are key players in this proposed roadmap. Other entities, including philanthropic foundations, international organizations, and venture capitalists, may also play important roles. Government agencies are responsible for policies and regulations, such as those on health insurance coverage of influenza vaccination cost and vaccine quality control and supply (red solid arrows, Figure 1). They also provide funding for education as well as innovation and research (red dotted arrows). Academic institutions and health‐care providers play a direct role in education (educating primary‐care physicians and other health‐care workers as well as the public) as well as innovation and research (blue solid arrows). With their expertise and leading roles in innovation and research, they may also influence policy‐making and vaccine supply (blue dotted arrows). Likewise, the vaccine manufacturing industry plays a role directly in vaccine supply as well as innovation and research (black solid arrows), and perhaps also indirectly in providing information on the influenza vaccines they manufacture to health‐care providers as well as the public (education, black dotted line). Other entities can directly play a role in education and innovation and research (green solid lines) and vaccine development and supply (green dotted arrow). Working together, it is hoped that these key players along with the public will improve policy, education, vaccine supply, as well as innovation and research, ultimately leading to universal influenza vaccination coverage for seniors and those who need vaccine protection in China.

## CONFLICTS OF INTEREST

Nothing to disclose.
